# Aggregation-prone GFAP mutation in Alexander disease validated using a zebrafish model

**DOI:** 10.1186/s12883-017-0938-7

**Published:** 2017-09-07

**Authors:** So-Hyun Lee, Tai-Seung Nam, Kun-Hee Kim, Jin Hee Kim, Woong Yoon, Suk-Hee Heo, Min Jung Kim, Boo Ahn Shin, Ming-Der Perng, Hyon E. Choy, Jihoon Jo, Myeong-Kyu Kim, Seok-Yong Choi

**Affiliations:** 1 Department of Biomedical Sciences, Chonnam National University Medical School, Gwangju, 501-759 Republic of Korea; 2 Center for Creative Biomedical Scientists at Chonnam National University, Gwangju, Republic of Korea; 3 Department of Neurology, Chonnam National University Medical School, Gwangju, 501-759 Republic of Korea; 4 Department of Microbiology, Chonnam National University Medical School, Gwangju, Republic of Korea; 5 Department of Radiology, Chonnam National University Medical School, Gwangju, Republic of Korea; 6 Department of Biological Sciences, Sookmyung Women’s University, Seoul, Republic of Korea; 7 Institute of Molecular Medicine, College of Life Sciences, National Tsing Hua University, Hsinchu, Taiwan

**Keywords:** Alexander disease, Leukodystrophy, Glial fibrillary acidic protein, Rosenthal fibers, Astrocyte, Zebrafish, GFAP

## Abstract

**Background:**

Alexander disease (AxD) is an astrogliopathy that predominantly affects the white matter of the central nervous system (CNS), and is caused by a mutation in the gene encoding the glial fibrillary acidic protein (GFAP), an intermediate filament primarily expressed in astrocytes and ependymal cells. The main pathologic feature of AxD is the presence of Rosenthal fibers (RFs), homogeneous eosinophilic inclusions found in astrocytes. Because of difficulties in procuring patient’ CNS tissues and the presence of RFs in other pathologic conditions, there is a need to develop an in vivo assay that can determine whether a mutation in the *GFAP* results in aggregation and is thus disease-causing.

**Methods:**

We found a *GFAP* mutation (c.382G > A, p.Asp128Asn) in a 68-year-old man with slowly progressive gait disturbance with tendency to fall. The patient was tentatively diagnosed with AxD based on clinical and radiological findings. To develop a vertebrate model to assess the aggregation tendency of GFAP, we expressed several previously reported mutant GFAPs and p.Asp128Asn GFAP in zebrafish embryos.

**Results:**

The most common GFAP mutations in AxD, p.Arg79Cys, p.Arg79His, p.Arg239Cys and p.Arg239His, and p.Asp128Asn induced a significantly higher number of GFAP aggregates in zebrafish embryos than wild-type GFAP.

**Conclusions:**

The p.Asp128Asn GFAP mutation is likely to be a disease-causing mutation. Although it needs to be tested more extensively in larger case series, the zebrafish assay system presented here would help clinicians determine whether GFAP mutations identified in putative AxD patients are disease-causing.

## Background

Alexander disease (AxD) is a neurodegenerative disorder that primarily affects the white matter of the central nervous system (CNS) [1–5]. It was first reported in 1949 by W. Stewart Alexander in a 15-month-old boy with megalencephaly, hydrocephalus and psychomotor retardation. The brain pathology of the boy showed “progressive fibrinoid degeneration of fibrillary astrocytes,” [6] which was later identified as Rosenthal fibers that were initially described by Werner Rosenthal in ependymoma in 1898 [7]. Rosenthal fibers are homogeneous eosinophilic inclusions stained by hematoxylin and eosin, and consist mainly of glial fibrillary acidic protein (GFAP), αB-crystallin, heat shock protein (HSP) 27 and cyclin D2 [2, 3, 5]. Messing and colleagues reported that AxD was elicited by mutations in the gene encoding GFAP, a type III intermediate filament predominantly found in astrocytes. They suggested that the mutations act in a gain-of-function fashion based on their finding that the phenotypes of *Gfap* null mice did not parallel those of AxD [8]. Since then, many different *GFAP* mutations have been reported in AxD patients [9].

AxD has been classified into three clinical subtypes depending on age at onset (AAO). Infantile AxD (birth to 2 years), the most frequent subtype, is characterized by progressive megalencephaly and/or hydrocephalus, developmental delay, psychomotor retardation, epileptic seizures. Juvenile AxD (2–14 years) features spastic paraplegia, progressive bulbar signs and ataxia with spared cognitive function. Adult AxD (late adolescence and beyond), the least frequent subtype and often misdiagnosed with multiple sclerosis, shows variable manifestations including progressive ataxia, tetraparesis, bulbar and pseudobulbar signs [3, 10]. A revised classification system was proposed based on statistical analysis of clinical, radiologic, and genetic features of 215 cases of AxD. In the revised system, patients with type I AxD show early AAO, macrocephaly, developmental delay and typical brain magnetic resonance imaging (MRI) features. In contrast, patients with type II AxD exhibit various AAO, bulbar symptoms, ocular movement abnormality and atypical MRI findings [11].

Although AxD can be diagnosed based on comprehensive evaluation of patient history, physical examination, brain MRI, *GFAP* sequencing and cerebral biopsy, *GFAP* sequencing and cerebral biopsy remain to be the best diagnostic approaches [3, 10]. Detection of Rosenthal fibers through cerebral biopsy is considered to be one of the best diagnostic approaches. However, most putative AxD patients with *GFAP* mutations did not undergo cerebral biopsy [12–14] as it is an invasive procedure. In addition, Rosenthal fibers are not a pathognomonic feature of AxD because they are also occasionally found in astrocytic tumors, ependymoma, hamartomas, craniopharyngioma, pineal cysts, glial scars and multiple sclerosis [3, 15]. Hence, DNA sequencing is the only definitive diagnostic approach for AxD under most circumstances. However, identification of GFAP mutations in putative AxD patients does not guarantee that these mutations are associated with AxD because it is feasible that these mutations are just variants of unknown significance. Therefore, it is imperative to determine whether the *GFAP* mutations found in tentative AxD patients are disease-causing. To this end, two methods have been employed. First, an in vitro assembly assay was performed with recombinant mutant GFAPs purified from *E. coli* and the formation of aggregates was then assessed. Second, an expression plasmid encoding the mutant *GFAP* was transfected into various mammalian cell lines, which were then observed for GFAP aggregates [13, 16–20]. However, these methods might not be suitable for testing the causality of the *GFAP* mutations, because both methods do not reflect the in vivo environment around astrocytes and the second method adopts a strong exogenous promoter to express mutant GFAP.

**Fig. 1:**
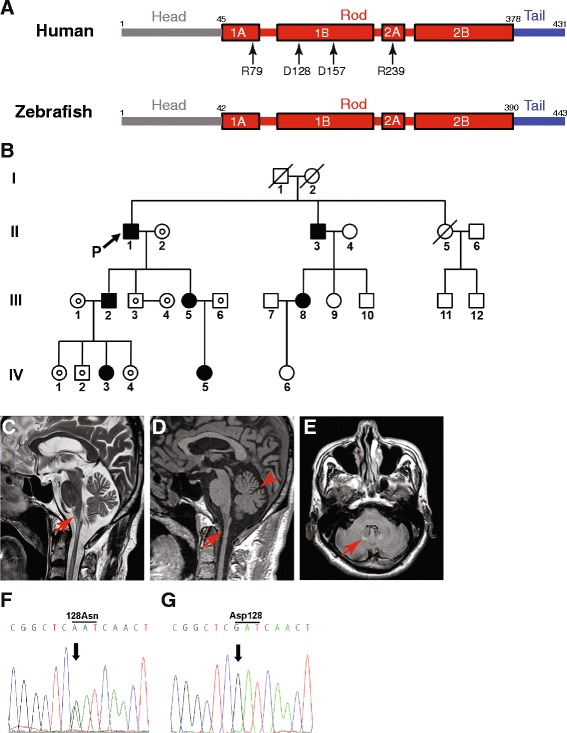
Clinical features and *GFAP* sequences of the proband. **a** Comparison between human and zebrafish GFAP, and location of amino acid residues whose mutations are discussed in this study. Human GFAP: NCBI accession number NP_002046; zebrafish Gfap: NP_571448. D: aspartate; R: arginine. **b** Pedigree of individuals with p.Asp128Asn GFAP shown as solid symbols. Symbols and nomenclature follow established guidelines [44]. A *small circle* within a square or a circle indicates an individual who tested negative for a *GFAP* mutation. P, proband. **c-e** Brain MR images of the proband. **c** Sagittal T2-weighted MR image shows marked atrophy of the medullar oblongata (*arrow*). **d** Sagittal T1-weighted MR image reveals prominent atrophy in the upper cervical cord (*arrow*) and cerebellar hemisphere (*arrowhead*). **e** Fluid-attenuated inversion recovery (FLAIR) image shows high signal intensity lesions in the bilateral cerebellar dentate nuclei (*arrow*). **f** and **g** DNA sequence analysis of the *GFAP*. *Arrows* indicate c.382G. **f** Electropherogram of the proband reveals a heterozygous G-to-A substitution at position 382 of the *GFAP*, which is predicted to substitute asparagine for aspartic acid (p.Asp128Asn). **g** Representative electropherogram of *GFAP* sequences in 200 control subjects

Zebrafish (*Danio rerio*) are tropical freshwater fish and a vertebrate model organism that is used to study vertebrate development because of transparent embryos, and rapid and external development. Especially, zebrafish have been extensively used to research nervous system development and to establish vertebrate models of neurodegenerative diseases [21, 22]. Zebrafish have astrocytes [23], and zebrafish Gfap shares 67% identity and 77% similarity with human GFAP, along with well-conserved hot spot amino acids mutated in AxD (Fig. 1a) [24]. In addition, regulatory elements that drive the specific expression of zebrafish *gfap* in astrocytes were identified [25].

We saw a patient who presented with slowly progressive gait disturbance and a missense mutation in the *GFAP*, and made a tentative diagnosis of AxD based on clinical and radiological findings. To determine whether the mutation is disease-causing, we set out to develop a zebrafish model that would be useful for molecular diagnosis of AxD.

## Methods

### Reagents

All chemicals were purchased from Sigma (St. Louis, MO), unless indicated otherwise.

### DNA sequencing

Genomic DNA (gDNA) was extracted from the peripheral blood of subjects using a Wizard Genomic DNA purification kit (Promega, Madison, WI), and all nine exons and exon–intron boundaries of the *GFAP* were PCR-amplified from the extracted gDNA as described previously [20, 26].

### DNA manipulation

**Fig. 2:**
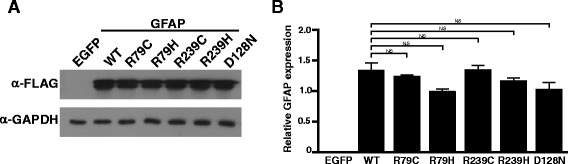
Protein expression levels of mutant alleles were comparable to that of WT GFAP. **a** HEK293T cells were transfected with plasmid encoding EGFP or indicated alleles of GFAP C-terminally fused to a FLAG epitope, and processed for Western blotting with anti-FLAG antibody. Anti-GAPDH (glyceraldehyde-3-phosphate dehydrogenase) antibody was used as a loading control. **b** Quantitation of GFAP band intensity in (**a**) normalized to GAPDH band intensity (*n* = 3). NS: not significant

For the expression study, human *GFAP* was PCR-amplified from *GFAP* cDNA (NCBI accession number BC013596, Dharmacon, Lafayette, CO) with specific primers (Table 1), and the resulting PCR product was cloned into the BamHI/EcoRV sites of the pCS4 + −3xFLAG-P2A vector [27]. p.Arg79Cys, p.Arg79His, p.Arg239Cys, p.Arg239His and p.Asp128Asn mutations were individually inserted into the WT *GFAP* construct by site-directed mutagenesis with specific primers (Table 1). For zebrafish study, the zebrafish *gfap* regulatory elements (7.4 kb) [25] were cloned into the BglII/SalI sites of a mini-*Tol2* (T2AL200R150G) plasmid [28]. EGFP and human *GFAP* C-terminally fused to a FLAG epitope were then sequentially cloned into the resulting construct (Fig. 2b). All plasmids constructed were verified by DNA sequencing (Macrogen, Daejeon, Korea).

**Table 1:** Sequences of primers (5′ → 3′) used to construct plasmids encoding various human *GFAP* alleles

Allele	Sequences
WT	Forward: TAGTAGGATCCATGGAGAGGAGACGCATCAC
	Reverse: TAGTCGATATCATCATCACATCCTTGTGCTCCTGCTTG
p.Arg79Cys	Forward: GAGATGATGGAGCTCAATGACtGCTTTGCCAGCTACATCGAG
	Reverse: CTCGATGTAGCTGGCAAAGCaGTCATTGAGCTCCATCATCTC
p.Arg79His	Forward: GAGATGATGGAGCTCAATGACCaCTTTGCCAGCTACATCGAG
	Reverse: CTCGATGTAGCTGGCAAAGtGGTCATTGAGCTCCATCATCTC
p.Asp128Asn	Forward: GAGAGCTGCGGCTGCGGCTCaATCAACTCACCGCCAACAG
	Reverse: CTGTTGGCGGTGAGTTGATtGAGCCGCAGCCGCAGCTCTC
p.Asp157Asn	Forward: GCAGAAGCTCCAGaATGAAACCAACCTG
	Reverse: CAGGTTGGTTTCATtCTGGAGCTTCTGC
p.Arg239Cys	Forward: CAGCCCTGAAAGAGATCtGCACGCAGTATGAGGCAATG
	Reverse: CATTGCCTCATACTGCGTGCaGATCTCTTTCAGGGCTG
p.Arg239His	Forward: CAGCCCTGAAAGAGATCCaCACGCAGTATGAGGCAATG
	Reverse: CATTGCCTCATACTGCGTGtGGATCTCTTTCAGGGCTG

Lower case indicates mutated nucleotides

### Cell culture and western blotting

HEK293T cells were purchased from American Type Culture Collection (Manassas,VA), cultured in Dulbecco’s modified Eagle’s media (Welgene, Daegu, Korea) supplemented with 10% fetal bovine serum (Thermo Fisher Scientific Korea, Seoul, Korea), and transfected with plasmid using Lipofectamine 2000 (Thermo Fisher Scientific Korea) according to the manufacturer’s instructions. Subsequently, the cells were lysed with M-PER mammalian protein extraction reagent (Thermo Fisher Scientific Korea) at 48 h post-transfection, and processed for Western blotting as described previously [27]. The antibodies used were anti-FLAG antibody (1:2000, Sigma-Aldrich, catalog number F1804), anti-glyceraldehyde-3-phosphate dehydrogenase (anti-GAPDH) antibody (1:2000, Trevigen, Gaithersburg, MD, 2275-PC-100), HRP-conjugated goat anti-mouse antibody (1:4000, Santa Cruz Biotechnology, Dallas, TX, sc-2005), and HRP-conjugated goat anti-rabbit antibody (1:4000, Santa Cruz Biotechnology, sc-2004). Band intensity on the Western blots was analyzed using ImageJ.

### Zebrafish study

Wild-type (WT) zebrafish (AB strain) were obtained from the Zebrafish International Resource Center (Eugene, OR), maintained using standard procedures [29] and staged in hours post-fertilization (hpf) as per standard criteria [30]. One-cell stage zebrafish embryos were microinjected with GFAP expression constructs (50 pg), anesthetized at 30 hpf in 0.02% tricane, mounted with 3% methylcellulose and imaged with an LSM 510 CLM (Zeiss, Hamburg, Germany). Z-series of images (15 images; interval thickness: 1.0 μm) were collected and presented as a stacking image. Resulting images were assembled using Adobe Photoshop (San Jose, CA), and aggregations were counted blindly.

### Statistical analysis

*P* values [31] were determined with the two-tailed paired Student’s t test. *P* < 0.05 was considered statistically significant.

### Transmission electron microscopy (TEM)

TEM was performed in the Electron Microscopy Facility at Yonsei Biomedical Reseach Institute at Yonsei University College of Medicine. In brief, zebrafish embryos injected with expression plasmids encoding WT or p.Arg79Cys GFAP were fixed at 30 hpf in 0.1 M phosphate buffer (pH 7.4) with 2% glutaraldehyde (Merck, Darmstadt, Germany) and paraformaldehyde (Merck) for 12 h, washed in 0.1 M phosphate buffer, post-fixed with 1% OsO_4_ in 0.1 M phosphate buffer for 90 min, dehydrated with an ascending ethanol series (50%, 60%, 70%, 80%, 90%, 95% and 100%) for 10 min each, and infiltrated with propylene oxide for 10 min. Subsequently, specimens were embedded with a Poly/Bed 812 embedding kit (Polysciences, Warrington, PA), polymerized in an electron microscope oven (TD-700, DOSAKA, Kyoto, Japan) at 65 °C for 12 h, cut into 200 nm thick semi-thin sections using an EM UC7 ultramicrotome (Leica Microsystems, Wetzlar, Germany) with a diamond knife (DiATOME, Hatfield, PA), stained with toluidine blue and observed with a light microscope. The region of interest was then cut into 80 nm thick ultra-thin sections using the ultramicrotome, placed on copper grids, stained in 4% uranyl acetate (Electron Microscopy Sciences, Hatfield, PA) for 20 min followed by lead citrate (Thermo Fisher Scientific Korea) for 10 min, and imaged with a transmission electron microscope (JEM-1011, JEOL, Tokyo, Japan) equipped with a MegaView III CCD camera (Olympus Soft Imaging Solutions, Lakewood, CO) at the acceleration voltage of 80 kV.

## Results

### A 68-year-old male with ataxia

A 68-year-old Korean man (proband; subject II.1 in Fig. 1b), who exhibited slowly progressive gait disturbance with tendency to fall for several months, was referred to our hospital. Albeit self-ambulatory, the proband suffered from unsteady gait due to ataxia. His medical history was unremarkable except for mild hypertension. The proband had no family history of neurological diseases or consanguineous marriage. Neurological examination revealed dysphagia, dysarthria, dysphonia, wide-based truncal ataxia, bilateral gaze-evoked nystagmus and exaggerated deep tendon reflexes with bilaterally positive Babinski and Hoffman signs, indicating dysfunctions in the brainstem, cerebellum or cervical cord. The proband did not present with sensory disturbance, palatal myoclonus, and abnormal mental or emotional status. To determine the etiology of ataxia, extensive workup was carried out including somatosensory evoked potential recording, blood and urine chemistry tests, assay of serum levels of vitamin B12 and thyroid hormones, venereal disease research laboratory (VDRL) test, anti-human immunodeficiency virus (HIV) antibody test and genetic studies for spinocerebellar ataxia (types 1, 2, 3, 6, 7 and 8), Friedreich ataxia and dentato-rubro-pallido-luysian atrophy. All of these tests were negative, however.

The brain MR images revealed marked atrophy of the medulla oblongata and upper cervical cord, and mild atrophy of the cerebellar hemisphere on both sagittal T2- and T1-weighted images (Fig. 1c and d, respectively). Moreover, fluid-attenuated inversion recovery (FLAIR) imaging illustrated hyperintense lesions in the bilateral cerebellar dentate nuclei (Fig. 1e). These MRI findings suggested adult AxD. Therefore, we had the proband’s *GFAP* sequenced and found a heterozygous mutation, c.382G > A, which was absent in the *GFAP* sequences from 200 normal controls. This substitution was predicted to lead to p.Asp128Asn (Fig. 1f and g). Sequencing of the *GFAP* of the proband’s family members suggested Mendelian inheritance of the mutation (Fig. 1b). Out of the other family members with p.Asp128Asn, subjects II.3, III.2, and III.5 showed hyperreflexia of the upper and lower extremities with positive Babinski and Hoffman signs and without evidence of neurological symptoms (Fig. 1b), indicating early stage of adult onset AxD or various degrees of penetrance.

p.Asp128Asn was reported in two cases of AxD, and Rosenthal fibers in the brain were observed posthumously in one of the cases [32, 33]. Albeit characteristic, Rosenthal fibers are not pathognomonic of AxD [3, 15]. As such, we turned to a zebrafish model to test if p.Asp128Asn GFAP is disease-causing.

### Zebrafish can be used to assess the formation of mutant GFAP aggregates

**Fig. 3:**
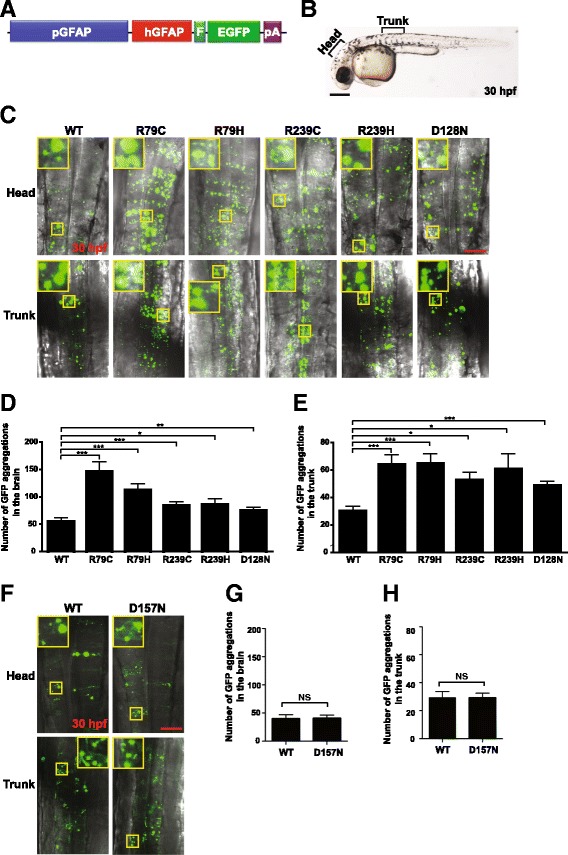
Aggregation susceptibility of mutant GFAPs can be assessed using zebrafish**. a** Schematic representation of an expression plasmid encoding human GFAP C-terminally fused to a FLAG epitope and EGFP driven by a zebrafish *gfap* promoter. EGFP: enhanced green fluorescent protein; F: 3× FLAG epitope tag; hGFAP: human GFAP; pA: polyadenylation sequence; and pGFAP: zebrafish *gfap* promoter. **b** Regions of zebrafish embryos at 30 h post-fertilization (hpf) imaged in (**c**). **c** One-cell stage zebrafish embryos were microinjected with expression plasmids encoding WT or indicated alleles of GFAP and imaged with a confocal laser microscope at 30 hpf. Images represent stacking of Z-series of images. Insets represent magnifications of the boxed areas. R79C: p.Arg79Cys; R79H: p.Arg79His; R239C: p.Arg239Cys; R239H: p.Arg239His; and D128N: p.Asp128Asn. Scale bar = 150 μm. **d** and **e** GFP aggregates, indicated by *green dots*, were counted in the brain (**d**) and trunk (**e**) regions of each group in (**c**). n = WT: 10; R79C: 9; R79H: 12; R239C: 15; R239H: 8; and D128N: 11. *: *P* < 0.05; **: *P* < 0.01; ***: *P* < 0.001. **f** Aggregation assays were performed with WT or D157N allele of GFAP as described in (**c**). Insets represent magnifications of the boxed areas. D157N: p. Asp157Asn. **g** and **h** GFP aggregates were counted as described in (**d** and **e**). NS, not significant. Scale bar = 150 μm

Of mutant GFAPs reported in AxD patients, the most common are p.Arg79Cys, p.Arg79His, p.Arg239Cys and p.Arg239His [9]. To test if these mutant GFAPs aggregate in zebrafish embryos, we first generated expression plasmids individually encoding WT or one of the four GFAP mutants C-terminally fused to a FLAG epitope, and compared their expression levels in human embryonic kidney HEK293T cells by Western blotting. Expression levels of all of the mutants were comparable to that of WT GFAP (Fig. 2a and b), indicating that the four mutation alleles do not affect GFAP stability. We next individually cloned the WT or mutant alleles of GFAP C-terminally fused to a FLAG epitope and enhance green fluorescent protein (EGFP) into the 3′ end of the zebrafish *gfap* promoter [25], and the resulting constructs (Fig. 3a) were microinjected into one-cell stage zebrafish embryos. Subsequently, brain and trunk regions of the embryos expressing comparable levels of GFP at 30 hpf were imaged with a confocal laser microscope (CLM; Fig. 3b). Embryos microinjected with WT GFAP plasmids showed GFP aggregations. This was not surprising as supplementation of human GFAP to zebrafish that have their own GFAP proteins expressed, could lead to GFAP aggregation zebrafish. This is supported by the previous report that expression of WT human GFAP in mouse triggered aggregation of GFAP [34]. Nevertheless, the number of aggregations was significantly higher in both the head and trunk regions of the embryos microinjected with plasmids encoding common GFAP mutants (Fig. 3c, d, and e). To further validate this method as a tool to determine pathogenicity of GFAP mutations, we repeated the experiment with p.Asp157Asn GFAP that was previously reported to be a non-disease causing variant [13]. As expected, so significant difference in aggregation was noted between WT and p.Asp157Asn GFAP (Fig. 3f-h).

**Fig. 4:**
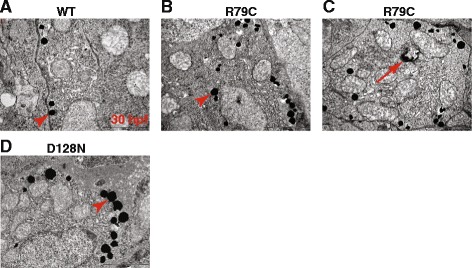
Aggregation susceptibility of mutant GFAPs can be assessed using zebrafish. **a**-**d** Zebrafish embryos at one-cell stage were microinjected with expression plasmids encoding WT (**a**), p.Arg79Cys GFAP (**b** and **c**), or p.Asp128Asn (**d**), and imaged at 30 hpf with transmission electron microscopy. *Arrows* and *arrowhead* indicate electron dense inclusions and a spherical structure with double-layered membranes, respectively. Scale bar = 2 μm

To check if GFP aggregations in zebrafish embryos are akin to GFAP aggregations in AxD patients, we performed transmission electron microscopy (TEM) on zebrafish embryos microinjected with expression plasmids encoding WT or p.Arg79Cys allele of GFAP, and indeed found electron dense inclusions in the cells of both groups of embryos (Fig. 4a-c), which is reminiscent of TEM findings of RFs in the astrocytes of the AxD brain [35]. Of note, more inclusions were observed in the TEM images of p.Arg79Cys embryos than WT embryos, consistent with CLM images. Intriguingly, found in the p.Arg79Cys embryos were the spherical structures with double layer membranes containing electron dense inclusions (Fig. 4c). These were reminiscent of autophagosome, which were previously reported in the AxD patient’s brain, mouse brain expressing p.Arg236His, and human astrocytoma U251 cells expressing p.Arg239Cys [35, 36]. Taken together, this outcome indicates that the GFAP aggregation assay in zebrafish embryos can be employed to assess the pathogenicity of GFAP mutations identified in patients tentatively diagnosed with AxD.

### P.Asp128Asn induces a significantly higher number of GFAP aggregations in the zebrafish embryos compared to WT GFAP

To ascertain whether the p.Asp128Asn GFAP allele of the proband is aggregation prone, we first compared expression levels between p.Asp128Asn GFAP and WT GFAP in HEK293T cells by Western blotting and observed comparable expression levels of the two GFAP alleles (Fig. 2a and b), demonstrating that the p.Asp128Asn does not affect GFAP stability. We next repeated the aggregation assay in zebrafish embryos with p.Asp128Asn GFAP plasmids, and found that p.Asp128Asn induced a significantly higher number of GFP aggregations in both head and trunk regions of the embryos compared to GFAP WT (Fig. 3c-e). Moreover, TEM revealed more electron dense inclusions in p.Asp128Asn embryos than WT embryos (Fig. 4a, d). These findings indicate that p.Asp128Asn is aggregation prone and thus may cause AxD.

## Discussion

Here, we show that a 68-year-old male with ataxia and atrophy of the medulla oblongata, upper cervical cord and the cerebellar hemispheres on brain MRI harbors a p.Asp128Asn GFAP mutation. Furthermore, we demonstrate that the p.Asp128Asn mutation induces more GFAP aggregations in zebrafish embryos than WT GFAP, suggesting that this mutation may cause AxD.

Most neurodegenerative diseases are protein-misfolding disorders (PMDs), and animal models of PMDs are instrumental in addressing many important questions about their molecular pathogeneses and the development of therapeutic modalities. Hence, several model organisms have been utilized to generate animal models of PMDs. For example, Hart and colleagues expressed polyglutamine tracts in the ASH sensory neurons of *Caenorhabditis elegans* to model Huntington’s disease and found neurodegeneration and apoptosis of ASH neurons [37]. Zhong and colleagues expressed amyloid-β peptides Aβ42 in the neurons of *Drosophila melanogaster* and noted amyloid deposits, late-onset progressive neurodegeneration and olfactory learning defects [38]. Ayyagari and colleagues showed that a mutant allele of asparaginase like-1 (ASRGL1) identified in a family with inherited retinal degeneration induced protein aggregation in monkey kidney fibroblast-like COS-7 cells, and retinal photoreceptor degeneration in zebrafish larvae [39]. Hsiao and colleagues generated Tg2576 transgenic mice expressing a Swedish allele of amyloid precursor protein and observed impaired learning and memory and the deposition of amyloid plaques in the brain [19]. Although invertebrate model organisms such as *C. elegans* and *D. melanogaster* have been used to model PMDs, they have certain limitations. First, they lack key factors critical to many human PMD pathogeneses, such as myelination, specialized neuronal and glial cell types, and a sophisticated immune system. Second, the anatomical structures of their brains are quite divergent from those of humans [40]. On the other hand, zebrafish are vertebrates such that zebrafish can overcome aforementioned limitations. In addition, zebrafish present other advantages as a model of PMDs, such as small size, transparency, and external embryonic development. Thus, zebrafish can be used not only to investigate the molecular pathogenesis of PMDs, but also to develop therapeutics against PMDs. For example, inhibitors of polyglutamine protein aggregation were identified using zebrafish embryos [41]. As such, the zebrafish GFAP aggregation model we present here will help elucidate the molecular pathogenesis of AxD and serve as a basis for the development of AxD therapeutics.

In the present study, plasmids encoding mutant GFAPs were injected into zebrafish embryos at 1 hpf, and GFP aggregates in the embryos were imaged at 30 hpf (Fig. 2d). This signifies that the zebrafish embryo assay system can determine aggregation tendency of mutant GFAPs in less than two days. Therefore, this system would be useful for clinicians to make a swift and accurate diagnosis of AxD.

We demonstrated in zebrafish embryos that the p.Asp128Asn induced less GFAP aggregates than p.Arg79Cys, p.Arg79His, p.Arg239Cys and p.Arg239His. The proband’s AxD appears to be classified as adult or type II AxD given the proband’s late AAO, bulbar symptoms, nystagmus and atypical MRI features [3, 10, 11]. Two previously reported cases of p.Asp128Asn also seem to be of the same classification [32, 33]. On the other hand, almost all cases of p.Arg79Cys, p.Arg79His, p.Arg239Cys and p.Arg239His fall under the infantile or type I AxD classification [11]. It is therefore tempting to speculate that the aggregation tendency of GFAP mutants may be related to AAO: high aggregation tendency results in early AAO, thereby infantile or type I AxD, and low aggregation tendency brings about late AAO, leading to adult or type II AxD. This notion is supported by a report by Perng and colleagues that two mutant alleles of GFAP found in infantile AxD, p.Asn386Ile and p.Asp417MetfsX14, induced more GFAP aggregates than did three mutant alleles found in adult AxD, p.Ser393Ile, p.Ser398Phe and p.Ser398Tyr [42]. Of course, this notion warrants further comprehensive investigation.

Messing and colleagues reported that p.Arg239His GFAP increased *Gfap* promoter activity in mice compared to WT GFAP [43]. This finding may be extended to other pathogenic GFAP mutations. If this is the case, higher aggregations we observed for pathogenic GFAP mutations might ensue from higher GFAP expression, at least in part. This notion warrants further investigation.

## Conclusions

Establishment of a zebrafish embryo assay system that could be employed to assess in vivo susceptibility of GFAP to aggregation, would help clinicians determine whether GFAP mutations identified in putative AxD patients are disease-causing.

## Abbreviations

AAO: Age at onset
AxD: Alexander disease
CLM: Confocal laser microscope
CNS: Central nervous system
EGFP: Enhance green fluorescent protein
FLAIR: Fluid-attenuated inversion recovery
gDNA: genomic DNA
GFAP: Glial fibrillary acidic protein
HIV: Human immunodeficiency virus
Hpf: Hours post-fertilization
HSP: Heat shock protein
MRI: Magnetic resonance imaging
PMD: Protein-misfolding disorder
RF: Rosenthal fiber
TEM: Transmission electron microscopy
VDRL: Venereal disease research laboratory
WT: Wild-type
